# SYMPTOM BURDEN, POSITIVE AFFECT AND RESILIENCY IN OLDER ADULTS EXPERIENCING NORMAL AGING

**DOI:** 10.1093/geroni/igad104.3613

**Published:** 2023-12-21

**Authors:** Reid Anctil, Angela Chang, Calli Mitchell, Lara Traeger, Elyse Park, Zeba Ahmad

**Affiliations:** Massachusetts General Hospital, Boston, Massachusetts, United States; Suffolk University, Boston, Massachusetts, United States; Massachusetts General Hospital, Boston, Massachusetts, United States; Massachusetts General Hospital, Boston, Massachusetts, United States; Massachusetts General Hospital, Boston, Massachusetts, United States; Massachusetts General Hospital, Boston, Massachusetts, United States

## Abstract

Older adults (age≥65) with multimorbid disease burden may be at chronic risk for bothersome symptoms, which can consequently reduce positive affect (e.g., feelings of excitement and enthusiasm). However, we hypothesized that individuals with more resiliency (the ability to “bounce back” from distress) may experience positive affect despite symptom burden. We conducted secondary analysis of baseline self-report survey data (collected between 8/2023-10/2023) from a prospective study of resiliency in older adults residing in nine continuing care retirement communities (CCRCs) across the U.S. Eligible participants lived independently in CCRCs and scored >11/15 on the five-minute Montreal Cognitive Assessment. Surveys assessed symptom burden (Condensed Memorial Symptom Assessment Scale), resiliency (Current Experience Scale), and positive affect (Positive and Negative Affect Schedule-Positive Affect). Bivariate correlations and multiple linear regression evaluated associations of symptom burden and resiliency with positive affect. Participants (M age =81, SD=5.6) were predominantly White (96%) and female (75%). Greater symptom burden correlated with lower positive affect (r=-.295, p<.001) and lower resiliency (r=-2.56, p<.001). In a multiple regression model, lower symptom burden (β = -.136, p=.003) and more resiliency (β=.626, p<.001) were independently associated with greater positive affect. The overall model accounted for 45.4% of variance in positive affect (F(2, 284) =117.198, p<.001). Lower symptom burden and more resiliency contributed to more positive affect among older adults in senior living communities. Because resiliency and positive affect are both potentially modifiable targets for intervention, longitudinal study and psychosocial interventions should explore the effects of increased resiliency on positive affect and general well-being over time.

